# Cross-sectional analysis of educational inequalities in primary prevention statin use in UK Biobank

**DOI:** 10.1136/heartjnl-2021-319238

**Published:** 2021-07-27

**Authors:** Alice Rose Carter, Dipender Gill, George Davey Smith, Amy E Taylor, Neil M Davies, Laura D Howe

**Affiliations:** 1 Medical Research Council Integrative Epidemiology Unit, University of Bristol, Bristol, UK; 2 Population Health Sciences, Bristol Medical School, University of Bristol, Bristol, UK; 3 Department of Epidemiology and Biostatistics, School of Public Health, Imperial College London, London, UK; 4 Clinical Pharmacology and Therapeutics Section, Institute of Medical and Biomedical Education and Institute for Infection and Immunity, St George's University Hospitals NHS Foundation Trust, London, UK; 5 Novo Nordisk Research Centre Oxford, Old Road Campus, Oxford, UK; 6 Clinical Pharmacology and Therapeutics Section, Institute of Medical and Biomedical Education and Institute for Infection and Immunity, St George’s, University of London, London, UK; 7 Clinical Pharmacology Group, Pharmacy and Medicines Directorate, St George’s University Hospitals NHS Foundation Trust, London, UK; 8 NIHR Bristol Biomedical Research Centre, Bristol, UK; 9 K.G. Jebsen Center for Genetic Epidemiology, Department of Public Health and Nursing, NTNU, Norwegian University of Science and Technology, Trondheim, Norway

**Keywords:** statins, epidemiology, electronic health records, risk factors

## Abstract

**Objective:**

Identify whether participants with lower education are less likely to report taking statins for primary cardiovascular prevention than those with higher education, but an equivalent increase in underlying cardiovascular risk.

**Methods:**

Using data from a large prospective cohort study, UK Biobank, we calculated a QRISK3 cardiovascular risk score for 472 097 eligible participants with complete data on self-reported educational attainment and statin use (55% female participants; mean age 56 years). We used logistic regression to explore the association between (i) QRISK3 score and (ii) educational attainment on self-reported statin use. We then stratified the association between QRISK3 score and statin use, by educational attainment to test for interactions.

**Results:**

There was evidence of an interaction between QRISK3 score and educational attainment. Per unit increase in QRISK3 score, more educated individuals were more likely to report taking statins. In women with ≤7 years of schooling, a one unit increase in QRISK3 score was associated with a 7% higher odds of statin use (OR 1.07, 95% CI 1.07 to 1.07). In women with ≥20 years of schooling, a one unit increase in QRISK3 score was associated with an 14% higher odds of statin use (OR 1.14, 95% CI 1.14 to 1.15). Comparable ORs in men were 1.04 (95% CI 1.04 to 1.05) for ≤7 years of schooling and 1.08 (95% CI 1.08, 1.08) for ≥20 years of schooling.

**Conclusion:**

Per unit increase in QRISK3 score, individuals with lower educational attainment were less likely to report using statins, likely contributing to health inequalities.

## Introduction

Despite reductions in cardiovascular disease (CVD) morbidity and mortality in high-income countries, the most socioeconomically deprived groups have the highest risk of disease.^1^ There is evidence that education is a causal risk factor for CVD.^2^

Previous studies have assessed the association of socioeconomic position (SEP) with primary and secondary treatment rates for statins with mixed results.^3–8^ Lower education is associated with higher levels of cardiovascular risk factors^2^ and therefore a greater underlying cardiovascular risk and clinical need for statins. However, educational differences in health-seeking behaviours or interactions between patients and clinicians, may mean patients with higher education are more likely to be prescribed statin medication.^9^ Independent of SEP, an overuse of statins in patients at low cardiovascular risk and underuse of statins in patients at high cardiovascular risk has been reported.^8 10^

Using UK Biobank, we investigated whether for a unit increase in QRISK3 cardiovascular risk score,^11^ participants with lower education were less likely to report taking statins for primary prevention than those with higher education. At the time of data collection (2006–2010), guidelines recommended prescribing statins to individuals with a ≥20% risk of experiencing an adverse cardiac event in 10 years, calculated using the Framingham risk score.^12^ In England and Wales, these guidelines have been updated to recommend prescribing based on a QRISK3 score of ≥10%.^13^ Cardiovascular risk assessments are typically carried out by a primary healthcare professional during routine health checks. Since 2004, low-dose statins have also been available to purchase over the counter from a pharmacy.

## Methods

### UK Biobank

At baseline, UK Biobank recruited 503 317 UK adults, aged 37–73 years, from 2006 to 2010. Participants attended assessment centres involving questionnaires, interviews, anthropometric and physical measurements.^14^ This analysis uses data from baseline assessments, linked hospital inpatient records and mortality statistics and linked primary care data (including prescriptions).

### QRISK score

**Figure 1:**
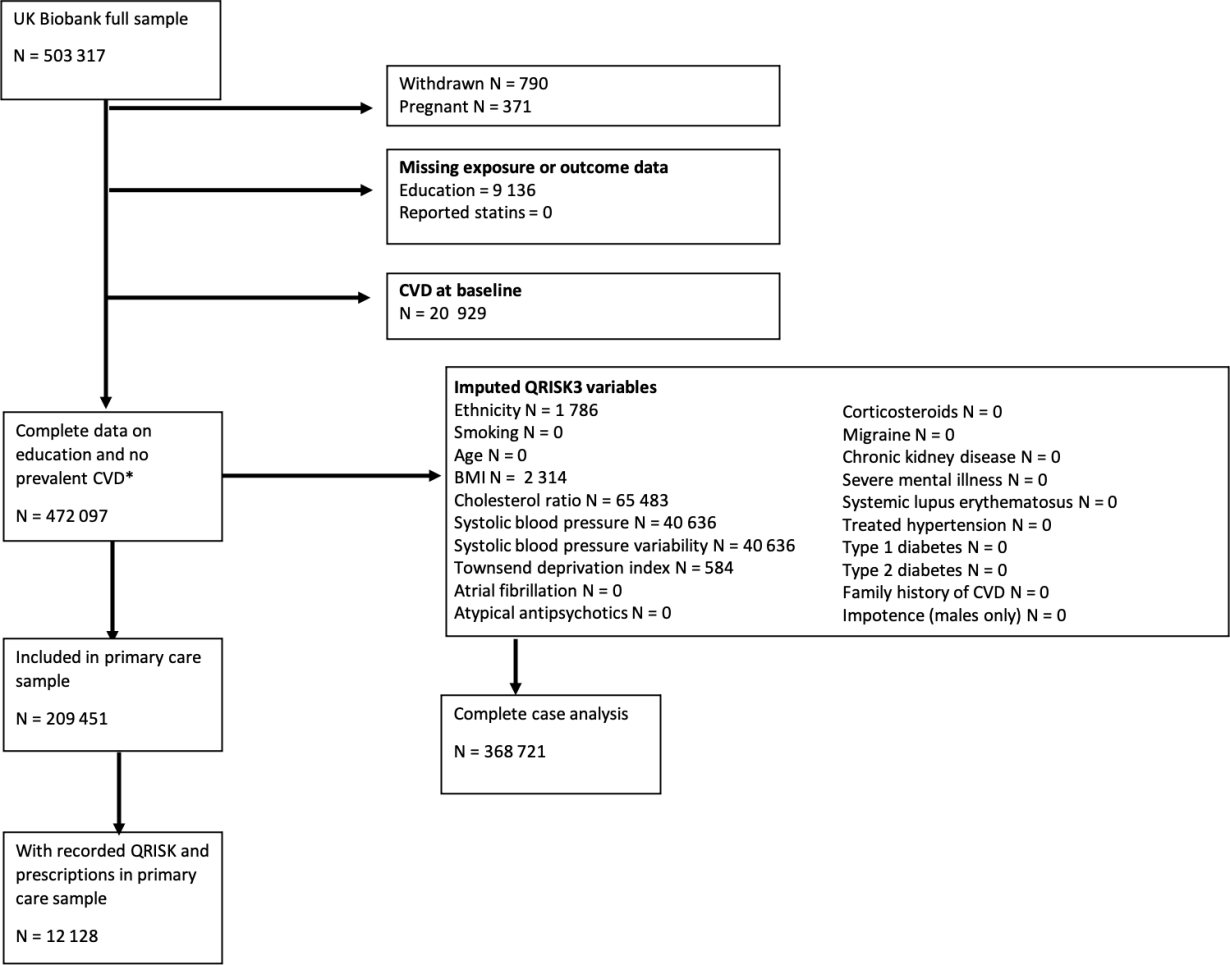
Study flow chart identifying eligible participants for analysis. BMI, body mass index; CVD, cardiovascular disease.

Cardiovascular risk was assessed using the publicly available QRISK3 algorithm (see https://qrisk.org/three/index.php).^11^ QRISK3 scores were derived for all participants with complete data on education, self-reported statin use and with no prevalent CVD (see exclusion criteria) (n=472 097) (figure 1). Multiple imputation was used for missing data in the QRISK3 variables (see ‘Statistical analyses’ section).

See online supplemental methods and online supplemental table 1 for full details of all QRISK3 variables and online supplemental tables 2 and 3 for UK Biobank treatment codes, International Classification of Diseases (ICD)-9 and ICD-10 codes used to define diagnoses.

10.1136/heartjnl-2021-319238.supp1 Supplementary data

In a subset of individuals with linked primary care data, QRISK (read 2 code: 38DF.) (n=1495), and QRISK2 scores (read 2 code: 39DP.) (n=10 633) were recorded from 2007 onwards. In sensitivity analyses, the first recorded QRISK score was used.

### Measuring education

Self-reported highest qualification was converted to the International Standard Classification for Education (ISCED) for years of education (online supplemental table 4).

### Measuring statin use

Regularly prescribed medication was reported to study nurses, which was used define (i) statin use and (ii) type of statin used (atorvastatin, simvastatin, fluvastatin, pravastatin and rosuvastatin).

In individuals with primary care data, self-reported statin use was validated by a statin prescription both 3 months before and 3 months after baseline. In sensitivity analyses using primary care QRISK scores, statin use was defined as any statin prescription after a QRISK score was recorded, excluding individuals who reported using statins at baseline.

### Exclusion criteria

Individuals were excluded if they had at least one diagnosis of myocardial infarction, angina, stroke, transient ischaemic attack, peripheral arterial disease, type 1 diabetes, chronic kidney disease or familial hypercholesterolaemia at baseline, as the National Institute for Health and Care Excellence guidelines state these diagnoses should result in a statin prescription,^13^ defined using ICD codes in hospital inpatient data (online supplemental table 3).

Complete case analyses were carried out on 368 721 individuals, with complete data on age, sex, education, self-reported statin use and all QRISK3 variables (online supplemental table 1 and figure 1).

### Code and data availability

The derived variables have been returned to UK Biobank. The code used to derive QRISK3 scores, and conduct analyses is available at github.com/alicerosecarter/statin_inequalities. All analyses were carried out in Stata V.16.1 (StataCorp, College Station, Texas, USA).

### Statistical analyses

To maximise power and potentially reduce bias, multivariable multiple imputation by chained equations^15^ was used to impute missing data in QRISK3 variables, assuming missing at random. The imputation sample was defined as all individuals with complete data on education and reported statin use. The proportion of missing data for each variable ranged from 0% to 15% (online supplemental table 5). Imputation was carried out within strata of education and sex to preserve interactions.^16^ A total of 25 imputed datasets were generated,^17^ each analysed individually with results combined according to Rubin’s rules.

Because the QRISK3 score is derived sex-stratified, analyses were carried out sex-stratified.^11^

To confirm the validity of the derived QRISK3 score, a univariable logistic regression model was used to assess the association between QRISK3 score and (i) statin use (as defined previously) and (ii) incident CVD (see online supplemental methods).

We estimated the association between years of education with (i) QRISK3 score (using linear regression) and (ii) statin use (using logistic regression).

#### Testing for interaction between QRISK3 score and education on statin use

Logistic regression was used to estimate the association of QRISK3 score with statin use, stratified by years of education, estimating multiplicative interactions (online supplemental figure 2, route 1). Analyses were adjusted for date of assessment to account for changes in statin prescribing guidelines during the recruitment period. No other covariates were adjusted for, assuming all relevant variables were incorporated into the QRISK3 score. Evidence of an interaction between QRISK3 score and years of education was evaluated in a linear model where the interaction term QRISK3×education was included.

#### Secondary analyses

Atorvastatin has greater efficacy than simvastatin but is more costly.^18^ To test whether educational inequalities are present in the statin type prescribed, we estimated the interaction between QRISK3×education with atorvastatin compared with simvastatin in statin users (online supplemental figure 1, route 2).

Analyses between QRISK3×education on statin use and type of statin were replicated using complete case data (online supplemental figure 1, routes 3 and 4).

Analyses were replicated in participants with linked primary care data using (i) baseline measures of QRISK3 and self-reported statin use (online supplemental figure 1, route 5), (ii) baseline measures of QRISK3 with validated statin use (online supplemental figure 1, route 6) and (iii) QRISK or QRISK2 score recorded in primary care data with statin prescriptions (online supplemental figure 1, route 7). Primary care QRISK scores were included if they were recorded on or prior to the date of first statin prescription, but time between both events was not accounted for.

Sensitivity analyses were carried out excluding participants who reported taking non-statin lipid-lowering therapies. Main analyses were also replicated on the additive scale for interaction.

Two further QRISK3 scores were derived using baseline data excluding (i) systolic blood pressure variability and (ii) family history of CVD from QRISK3 scores (see online supplemental methods). The pairwise correlation between scores with and without these variables was tested.

## Results

### UK Biobank sample

In primary analyses (n=472 097), 55% of participants were female with a mean age of 56 years. In female participants, the QRISK3 score implied a mean 10-year risk of a cardiovascular event of 6.9% (SD=5.5). In male participants, the QRISK3 score implied mean a 10-year risk of a cardiovascular event of 13.1% (SD=8.4). Participants were more likely to have completed ≥20 years of education (female=35%, male=38%) than ≤7 years of education (female=14%, male=14%); 10% of female participants and 17% of male participantss reported using statins (online supplemental table 6).

The distribution of variables was similar between the multiply imputed data, complete case data and the subset of participants with primary care data (online supplemental table 6).

### Association of QRISK3 score with statins and cardiovascular disease

**Figure 2:**
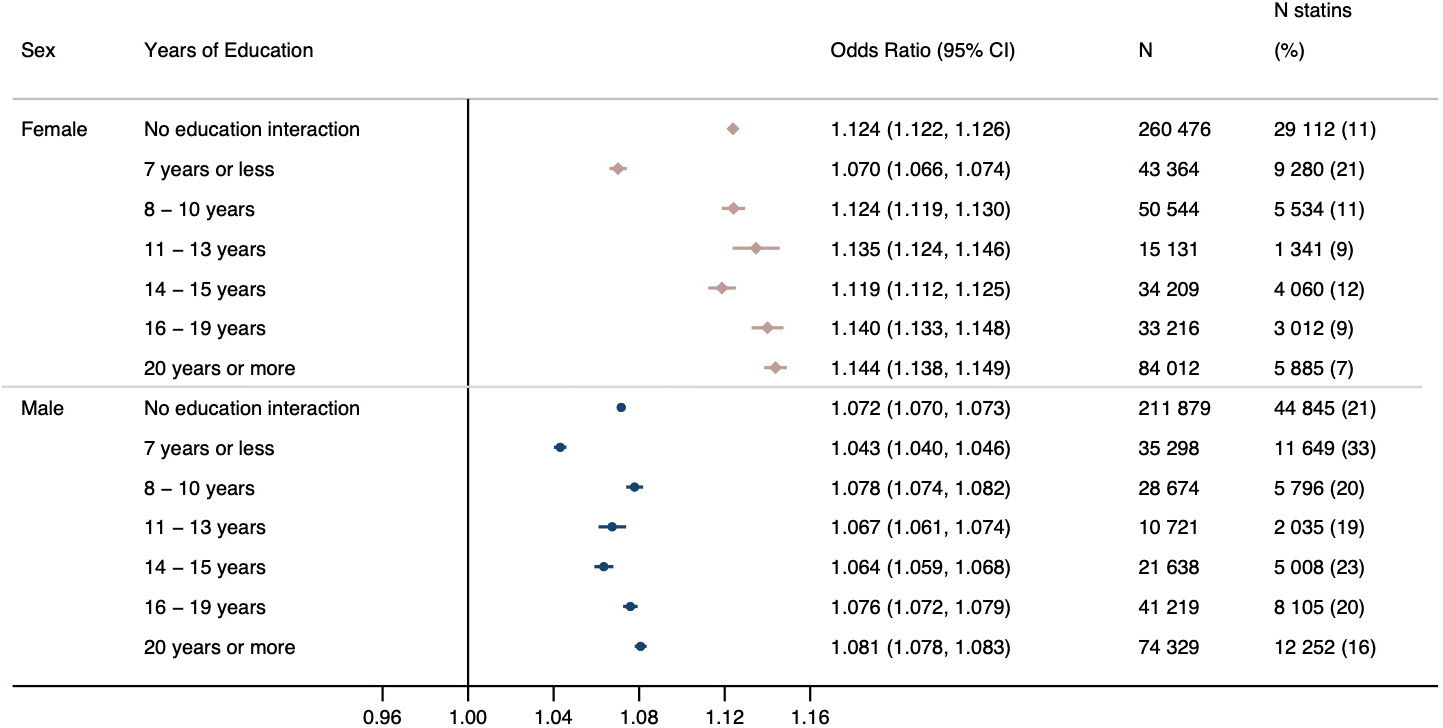
OR for self-reported statin use per unit increase in baseline QRISK3 score with no education interaction and stratified by years of education in female and male participants, adjusted for date of baseline assessment centre. Analyses stratified by years of education provide an estimate of interaction on the multiplicative scale. P value for interaction in female participants=1.896×10^−85^ and male participants=1.999×10^−48^.

Per one unit increase in QRISK3 score (ie, a 1% increase in the 10-year risk of experiencing a cardiovascular event) in female participants, the OR for statin use was 1.12 (95% CI 1.12 to 1.13) and the OR for incident CVD was 1.14 (95% CI 1.14 to 1.15) (figure 2, online supplemental figure 2 and online supplemental table 7). Female participants with a QRISK3 score of ≥10 were 1.34 times (95% CI 1.31 to 1.36) more likely to report using statins than those with a QRISK score <10. In male participants, the OR for statin use was 1.07 (95% CI 1.07 to 1.07) and 1.09 (95% CI 1.09 to 1.09) for incident CVD per unit higher QRISK3 score (figure 2, online supplemental figure 2 and online supplemental table 7). Male participants with a QRISK3 score of ≥10 were 1.49 times (95% CI 1.46 to 1.52) more likely to report using statins than those with a QRISK score <10. Participants reporting using statins had lower mean low-density lipoprotein cholesterol levels (the biological target of statins), compared with non-statin users (online supplemental figure 3).

### Association of education with QRISK3 score and statin use

Per year increase in education was associated with a −0.30 (95% CI −0.30 to −0.29) reduction in mean QRISK3 score in female participants and a −0.35 (95% CI −0.35 to −0.34) reduction in male participants (online supplemental table 8 and online supplemental figure 4).

Statin prevalence was highest in those with ≤7 years of education (equivalent to no formal qualifications) across all strata of cardiovascular risk (online supplemental figure 5 and online supplemental table 9). Each additional year of education was associated with a lower odds of statin use (OR in female participants: 0.93; 95% CI 0.93 to 0.93; OR in male participant: 0.96; 95% CI 0.96 to 0.96) (online supplemental figure 6).

### Interaction between education and QRISK3 score in relation to statin use

There was evidence of an interaction between QRISK3×education on statin use. In female participants, per unit increase in QRISK3, the OR for reporting statin use in those with ≥20 years (equivalent to obtaining a degree) was 1.14 (95% CI 1.14 to 1.15) compared with an OR of 1.07 (95% CI 1.07 to 1.07) for those with ≤7 years of education (figure 1). In male participants, the OR for statin use per unit increase in QRISK3 score in those with ≥20 years of education was 1.08 (95% CI 1.08 to 1.08) compared with an OR of 1.04 (95% CI 1.04 to 1.05) for those with ≤7 years (figure 2).

### Secondary analyses

There was little evidence of an interaction between QRISK3×education on statin type (online supplemental table 10 and online supplemental figure 7).

**Figure 3:**
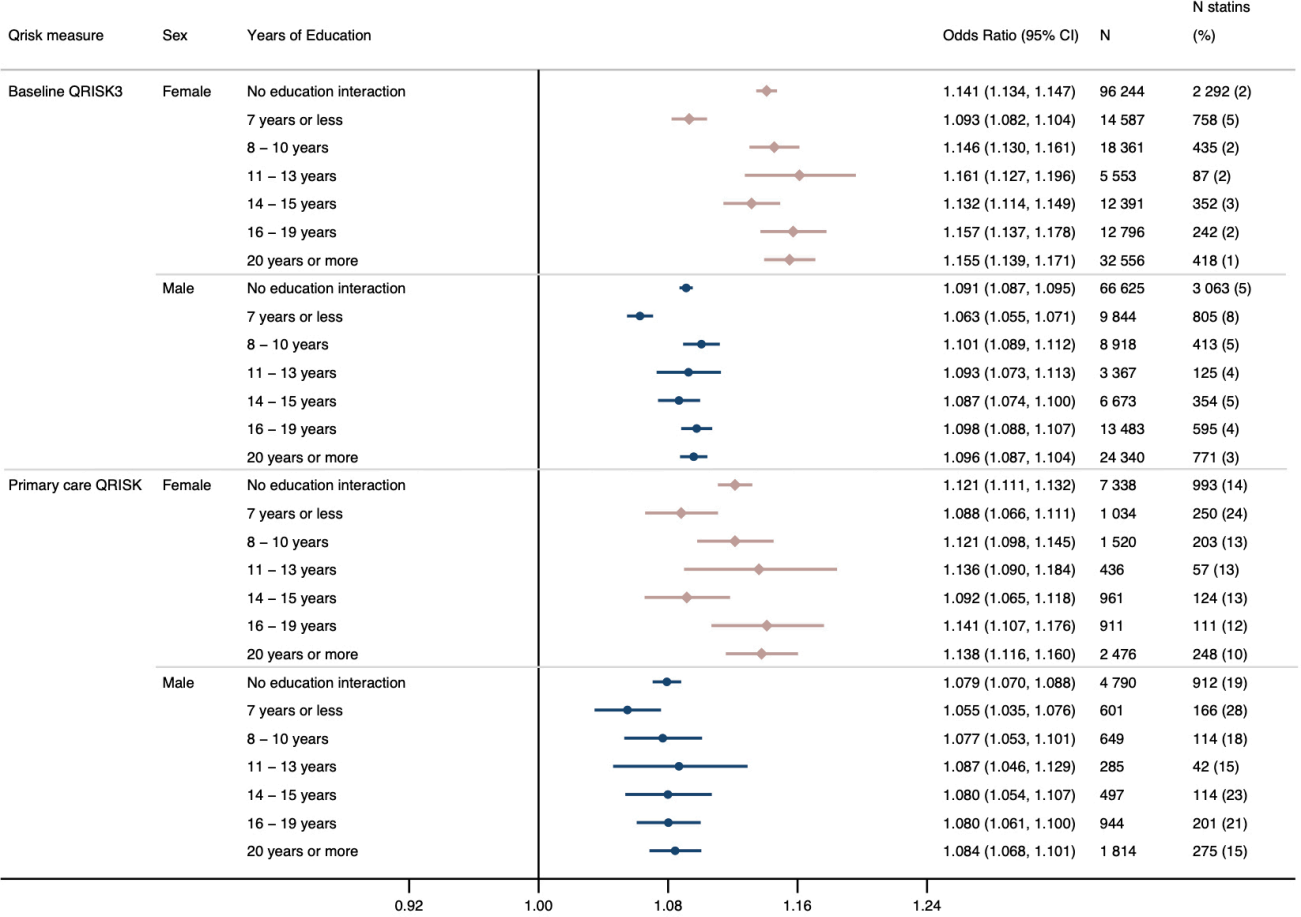
OR for statin use recorded in primary care prescription data per unit increase in (A) baseline QRISK3 score and (B) QRISK or QRISK2 score recorded in primary care, in female and male participants adjusted for date of baseline assessment centre or date of QRISK assessment in primary care. Analyses stratified by years of education provide an estimate of interaction on the multiplicative scale. Baseline QRISK3: p value for interaction in female participants=5.476×10^−10^ and male participants=4.046×10^−7^ QRISK score recorded in primary care: p value for interaction in female participants=0.006 and male participants=0.413.

In analyses in participants with primary care data using (i) baseline measures of QRISK3 and self-reported statin use, (ii) baseline measures of QRISK3 with prescription-validated statin use and (iii) QRISK or QRISK2 score recorded in primary care data with a statin prescription, similar interactions were observed to the main results, although evidence of an interaction was weaker in the primary care QRISK analyses in male participants (figure 3 and online supplemental figure 8).

Sensitivity analyses (i) using complete case data and (ii) excluding participants on non-statin-lowering therapy were consistent with the main results (online supplemental tables 11 and 12). There was evidence of an additive interaction between QRISK3×education, although the strength of the interaction was weaker compared with the multiplicative scale (online supplemental figure 9).

Pairwise correlation between the baseline-derived QRISK3 score and QRISK3 scores derived excluding (i) systolic blood pressure variability estimated from the difference between two baseline measures and (ii) self-report of any CVD in a mother, father or sibling, were high (all >0.97) (online supplemental table 13).

## Discussion

Despite a higher prevalence of statin use in less educated participants, these participants were less likely to receive statin treatment compared with more highly educated individuals given an equivalent increase in QRISK3 cardiovascular risk score.

### Results in context

Cardiovascular risk factors partly mediate the association between education and CVD^2 19–21^ and likely contribute to the greater clinical need for statins in individuals with lower education. However, differences in cardiovascular preventative medication may be further contribute to socioeconomic inequalities. We found the prevalence of statin use in participants at low cardiovascular risk (QRISK3 score of <10%) was similar to previous analyses in UK primary care databases.^10^ However, notably here, we found the prevalence of statin use in participants with low cardiovascular risk (<10% QRISK3) was higher in participants with lower educational attainment compared with higher educational attainment.

Since 2009, National Health Service health checks have been offered to English and Welsh residents aged 40–74 years without pre-existing conditions every 5 years, aiming to prevent a number of diseases including CVD.^22^ A recent systematic review identified seven studies illustrating inequalities in favour of those with higher SEP attending preventative health checks,^23^ including a trend towards lower uptake in smokers; a socially patterned cardiovascular risk factor.^23 24^ Increased engagement with preventative screening may reduce inequalities in CVD and statins. However, in analyses using QRISK scores and statin prescriptions recorded in primary care data, these inequalities remained. Therefore, health-seeking behaviours, including attending primary care clinics, cannot be the sole driver of inequalities.

Previous studies found mixed evidence for the association between SEP and statin use, including the direction of effect.^3–8^ However, there was often limited consideration for underlying cardiovascular risk.^3–6^ Forde *et al* adjusted for Framingham risk score to control for cardiovascular risk.^7^ In contrast to our results, they found no evidence of inequalities in statin use by strata of employment grade in the Whitehall II study. This difference could be due to different measures of SEP (education vs employment) or cohort differences, where the Whitehall II study is an occupational cohort. The QRISK score has also been shown to have a greater predictive power than the Framingham risk score.^25^ Therefore, our analyses may better account for cardiovascular risk.

In participants with primary care data, a large number of participants reported taking statins to study nurses but had no prescription at baseline. These individuals are potentially a combination of those purchasing statins over the counter, having a private prescription or no longer being prescribed statins. Most individuals (91%) without a linked prescription reported taking simvastatin (the only statin available over the counter). It is possible that accessing statins through private practices or over the counter are further contributing to inequalities in cardiovascular outcomes.

### Strengths and limitations

The major strength of our work is the large sample size and array of data available. Given the age of participants, statin prevalence is high. Using linked primary care data for 44% of the eligible sample we could (i) validate self-reported statin use and (ii) compare different mechanisms inequalities may arise. Where inequalities are present in primary care QRISK scores, inequalities are potentially due to factors within clinic settings. Using QRISK3 scores derived at baseline, inequalities may be due to differences in health-seeking behaviour.

Lifestyle and behavioural characteristics included in the QRISK3 score are likely measured more accurately in UK Biobank compared clinics. However, not all variables, or repeat measurements of variables specified in the QRISK3 algorithm are available in UK Biobank.^11^ The QRISK3 algorithm includes medications where an individual has two or more prescriptions for each class of medication (eg, corticosteroid or atypical antipsychotic). We relied on a single self-report measure at baseline, which may overestimate medication use. However, the magnitude to which these measurements differ is unlikely to introduce much bias to the QRISK3 score. Systolic blood pressure variability and coronary heart disease in a first-degree relative under the age of 60 years are not available in UK Biobank. Although we have included measures likely to capture some of these variables, this may introduce bias to the QRISK3 estimate.

Participants in UK Biobank are generally of a higher SEP and healthier than the general population, where higher education has been shown to increase participation and socially patterned cardiovascular risk factors including smoking decrease participation.^14 26^ Additionally, participants with lower SEP may differ from those of an equivalent SEP (or level of educational attainment) in the general population. Therefore, inequalities in the wider population may be greater than those reported here.

In these data, it is not possible to identify who has both received a prescription and subsequently had the prescription filled, for example, in primary analyses, individuals with the lowest levels of educational attainment may have received a prescription for a statin, but not collected the medication. This may explain why the interaction between QRISK3 scores, and educational attainment is larger in the analyses using self-reported statin use compared with statin prescriptions in primary care data.

We have used the ISCED definitions of education as a measure of SEP. Although education is a strong predictor of adulthood SEP, correlating with future employment and income, adult SEP may explain some of the non-linearities observed in these results.^27^

### Clinical implications

Our results indicate two potential mechanisms for these inequalities. First, there are likely to be differences in health-seeking behaviour.^28^ Second, there are important interactions between the healthcare practitioner and patient resulting in unequal prescribing of statins.

Given persisting inequalities in CVD, addressing the contribution of inequalities in statin prescribing provides a clear policy target. However, this requires systemic change and different interventions may be required to address the different mechanisms of inequalities. Future research should investigate what factors are driving inequalities, such as patient preference for treatment^29^ or non-up-take of preventative health checks.

## Conclusions

Our analyses demonstrate that for a unit increase in cardiovascular risk, individuals with lower levels of education are less likely to be prescribed statins compared with individuals with higher education, meaning differences in statin prescribing likely contribute to inequalities in CVD. Policies should consider how these inequalities can be minimised.

**Key messages**

**What is already known on this subject?**

- Despite reductions in the rates of cardiovascular disease in high-income countries, individuals who are the most socioeconomically deprived remain at the highest risk of disease.
- Although intermediate lifestyle and behavioural risk factors explain some of this, much of the effect remains unexplained.

**What might this study add?**

- Per unit increase in QRISK3 score, a measure of clinical need, the likelihood of statin use increased more in individuals with high educational attainment compared with individuals with lower educational attainment.
- These results were similar when using UK Biobank to derive QRISK3 scores and when using QRISK scores recorded in primary care records, and when using self-reported statin prescription data or prescription data from primary care records.

**How might this impact on clinical practice?**

- The mechanisms leading to these differences are unknown, but both health-seeking behaviours and clinical factors may contribute.
- Clinicians and policy makers should consider how they can improve uptake of preventative health checks to carry out cardiovascular risk assessments, while also considering whether any clinic-level factors could be addressed to improve the uptake of statins in patients with lower education.

## Data Availability

Data may be obtained from a third party and are not publicly available. The derived variables have been returned to UK Biobank for archiving. The code used to derive QRISK3 scores and carry out analyses is available at: github.com/alicerosecarter/statin_inequalities.
