# Supplementary material for: Cross-sectional analysis of educational inequalities in primary prevention statin use in UK Biobank
Source: Heart. 2021 Jul 27;108(7):536–42. doi: 10.1136/heartjnl-2021-319238 (PMC8921562; doi:10.1136/heartjnl-2021-319238)
Supplement: Supplementary data [file heartjnl-2021-319238supp001.pdf]

SUPPLEMENTAL MATERIAL

Supplementary Methods ... 3

UK Biobank ... 3

Variable definitions for use in QRISK3 scores ... 3

Diagnoses of disease ... 3

Treatments ... 3

Behavioral, lifestyle and biological factors ... 3

Ethnicity ... 3

Townsend deprivation index ... 3

BMI ... 3

Smoking ... 3

Biological factors ... 4

Systolic blood pressure ... 4

Systolic blood pressure variability ... 4

Total cholesterol:HDL cholesterol ratio ... 4

Coronary heart disease in a first degree relative under 60 years of age ... 4

Incident cardiovascular disease ... 4

Additional Tables ... 5

Supplementary Table 1: Variables used, and assumptions made when generating QRISK3 scores in UK Biobank participants at baseline ... 5

Supplementary Table 2: Treatment codes in UK Biobank to define medications ... 6

Supplementary Table 3: ICD codes used to define incident and prevalent cases of cardiovascular disease ... 8

Supplementary Table 4: International Standard for Classification of Education codes mapped to UK Biobank self-report highest qualification to estimate years of education ... 8

Supplementary Table 5: Proportion of missing data in QRISK3 variables ... 9

Supplementary Table 6: Descriptive characteristics of UK Biobank participants in i) the full eligible sample analysed ii) the full eligible sample who also have linked primary care data and iii) participants with linked primary care data and a recorded QRISK score ... 10

Supplementary Table 7: Odds of i) statin use and ii) incident cardiovascular disease per unit increase in QRISK3 score and unit increase in years of education, adjusted for date of baseline assessment ... 13

Supplementary Table 8: Mean difference in QRISK3 score per unit increase in educational attainment ... 13

Supplementary Table 9: Percent of participants reporting statin use in low, medium and high cardiovascular risk groups, stratified by years of education and the association between education and statin use stratified by cardiovascular risk ... 14

Supplementary Table 10: Odds of Atorvastatin use compared with Simvastatin (baseline) use per unit increase in QRISK3 score and by strata of educational attainment (not accounting for interactions), adjusted for date of baseline assessment ... 15

Supplementary Table 11: Odds of i) statin use and ii) Atorvastatin use compared with Simvastatin (baseline) use per unit increase in QRISK3 score stratified by educational attainment in the complete case sample to test for evidence of a multiplicative interaction......................................16

Supplementary Table 12: Odds of statin use per unit increase in QRISK3 score stratified by educational attainment in the complete case sample to test for evidence of an interaction, excluding participants on non-statin lipid-lowering therapies...17

Supplementary Table 13: Pairwise correlation for QRISK3 scores derived from baseline measures in UK Biobank including all variables and excluding i) family history of CVD and iii) systolic blood pressure variability 17

Additional Figures.. 18

Supplementary Figure 1: Schematic of primary and secondary analyses carried out.......................18

Supplementary Figure 2: Prevalence of statin prescribing by decile of QRISK3 score in females and males in individuals with complete data 19

Supplementary Figure 3: Mean level of low-density lipoprotein cholesterol by years of education in females and males stratified by self-report statin use 20

Supplementary Figure 4: Mean and median values of QRISK3 score on those with complete data, by years of education for females and males..21

Supplementary Figure 5: Prevalence of statin prescribing by years of education in females and males in individuals with complete data 22

Supplementary Figure 6: Odds ratio for statin use per year unit increase in educational attainment (all years) and per strata of educational attainment..23

Supplementary Figure 7: Odds ratio for Atorvastatin prescribing compared to Simvastatin, per unit increase in QRISK3 score with no education interaction and stratified by years of education in females and males to test for evidence of an interaction 24

Supplementary Figure 8: Odd ratio for self-report statin use per unit increase in baseline QRISK3 score with no education interaction and stratified by years of education to test for evidence of an interaction in the subsample of females and males with linked primary care data 25

Supplementary Figure 9 : Risk difference for self-report statin use per unit increase in baseline QRISK3 score with no education interaction and stratified by years of education in females and males to test for an interaction on the additive scale 26

Supplementary Methods

UK Biobank

All UK Biobank participants are linked to mortality records, hospital episode statistics (HES) or Scottish morbidity and mortality records (referred to jointly throughout as hospital admissions data), with data available from 1997 in England, 1998 in Wales and 1981 in Scotland, with the most recent entry recorded in this analysis in May 2017. A subset of participants (approximately 230,000) have linked primary care and prescribing data.

Variable definitions for use in QRISK3 scores

Diagnoses of disease

Diagnoses of disease including arthritis, diabetes (type I and type II), systemic lupus erythromatosus, atrial fibrillation, chronic kidney disease, migraine, HIV/AIDS, severe mental illness and erectile dysfunction were ascertained via linked hospital inpatients data or via linked medication data. All variables and assumptions made are available in Supplementary Tables 2-4.

Treatments

Use of drugs at baseline (antihypertensives, corticosteroids and atypical antipsychotics) were defined by self-reported medication use to clinic nurses at baseline. Individuals were coded as using medication if they reported any medication included in the QRISK3 score. In the QRISK3 derivation cohort individuals were required to have at least two prescriptions representing long term use. It was not possible to ascertain the number of prescriptions in UK Biobank; however, UK Biobank participants were asked to record regular treatments, rather than short term medication or over the counter medication. All treatment codes used to define these variables in UK Biobank are available in Supplementary Table 2.

Behavioral, lifestyle and biological factors

Ethnicity

Ethnicity was reported by participants to study nurses at UK baseline assessment centres. Ethnicity was categorised according to the categories used in the QRISK3 algorithm.

Townsend deprivation index

Townsend deprivation index of current location was recorded by UK Biobank at baseline .

BMI

Height (m) and weight (kg) were measured by UK Biobank study nurses ate baseline assessment centres which were used to calculate BMI (kg/m^2).

Smoking

Smoking status (never, former or current) was determined by self-reported data at baseline assessment centres. The number of cigarettes smoked per day in current smokers was reported at baseline assessment centres and categorised according to QRISK3 categories of light (1-9/day), moderate (10-19/day) and heavy smokers (≥20/day).

Biological factors

Systolic blood pressure

The mean from two resting automated measures of systolic blood pressure, measured using an Omron HEM-7105IT digital blood pressure monitor, was used in the QRISK3 score.

Systolic blood pressure variability

In the absence of repeated measures of systolic blood pressure on UK biobank a measure of systolic blood pressure variability was derived from the standard deviation of the two recorded measurements of systolic blood pressure at the baseline assessment centre.

Total cholesterol:HDL cholesterol ratio

Non-fasting measures of total serum cholesterol and high-density lipoprotein (HDL)-cholesterol were measured using enzymatic assays (Backman Coulter AU5800) and the ratio of the two values was calculated. UK Biobank corrected serum data for laboratory dilution effects and were excluded if they did not pass UK Biobank quality control.

Coronary heart disease in a first degree relative under 60 years of age

A measure of family history of cardiovascular disease was ascertained from reported heart disease in mothers, fathers and siblings of UK Biobank participants, however age of diagnosis, nor type of cardiovascular disease, could not be determined.

Incident cardiovascular disease

The validity of QRISK3 scores was assessed by evaluating the association between QRISK3 and incident cardiovascular disease (CVD) (see statistical analyses in main text). Incident CVD was defined using hospital admissions data. All cardiovascular subtypes were combined to define cases, and cases were any individual with an ICD10 I code or G45, or an ICD9 code between 3900-4599 recorded (see sTable 3). The follow up period was defined as any event following date of baseline assessment centre (between 2006 and 2010) until the most recent date available in the linked hospital inpatient data (May 2017).

Additional Tables

Supplementary Table 1: Variables used, and assumptions made, when generating QRISK3 scores in UK Biobank participants at baseline

Variable included in QRISK3 algorithm	Measured in UK Biobank by	ICD Code	UKBB Variable	Assumptions/limitations to the UK Biobank variables
**Diagnoses**				
Arthritis	Hospital inpatient data	M05		
Diabetes (Type I and II)	Hospital inpatient data	E10-E14		
Systemic lupus erythematosus	Hospital inpatient data	M32.9		
Atrial fibrillation	Hospital inpatient data	I48		
Chronic kidney disease	Hospital inpatient data	N18.3-N18.5		
Migraine	Hospital inpatient data	G43		
HIV/AIDS	Hospital inpatient data	B20		
Severe mental illness	Hospital inpatient data	F20, F23, F31, F32, F33		
Erectile dysfunction	Nurses interview treatment data	N52	n_20003_0	
**Treatments**				
Antihypertensives	Nurses interview treatment data		n_20003_0	Original QRISK3 derivation specifies that use of drugs at baseline was defined as at least two prescriptions, with the most recent one no more than 28 days before the date or cohort entry. This cannot be ascertained in UK Biobank baseline data
Corticosteroids	Nurses interview treatment data		n_20003_0	
Second generation atypical Psychotics	Nurses interview treatment data		n_20003_0	
**Lifestyle**				
Ethnicity	Self-report		n_21000_0_0	
Townsend deprivation index	Postcode at baseline		n_189_0_0	
BMI	Baseline clinic		n_21001_0_0	
Smoking	Self-report at baseline		n_20116_0_0<br>n_3456_0_0	Calculated from derived variable for cigarettes per day
**Biological Factors**				
Age	Baseline clinic		n_21003_0_0	
Systolic blood pressure	Baseline clinic		n_4080_0_1<br>n_4080_0_0	
Systolic blood pressure variability	Baseline clinic		n_4080_0_1n_4080_0_0	The QRISK3 algorithm uses the standard deviation of repeated values of blood pressure. This was not available in UK Biobank; therefore, systolic blood pressure variability was derived from the standard deviation between two baseline automated readings of systolic blood pressure
Total cholesterol: HDL ratio	Baseline clinic serum metabolomics		n_30690_0_0<br>n_30760_0_0	
Coronary heart disease in first degree relative (<60 years)	Self-report		n_20107_0_0<br>n_20110_0_0<br>n_20111_0_0	Includes all reported family history of CVD, not restricted to cases under 60 or specific subtypes

Supplementary Table 2: Treatment codes in UK Biobank to define medications

Medication	UK Biobank treatment code
Statins	1141146234 1140888594 1140888648 1141192410 1140861958
Erectile dysfunction	1140869100 1140883010 1141168936 1141168944 1141168946 1141168948 1141187810 1141187814 1141187818 1141192248 1141192256 1141192258 1141192260
Antihypertensives	1140860332 1140860334 1140860336 1140860338 1140860340 1140860342 1140860348 1140860352 1140860356 1140860358 1140860362 1140860380 1140860382 1140860386 1140860390 1140860394 1140860396 1140860398 1140860402 1140860404 1140860406 1140860410 1140860418 1140860422 1140860426 1140860434 1140860454 1140860470 1140860478 1140860492 1140860498 1140860520 1140860532 1140860534 1140860544 1140860552 1140860558 1140860562 1140860564 1140860580 1140860590 1140860610 1140860628 1140860632 1140860638 1140860654 1140860658 1140860690 1140860696 1140860706 1140860714 1140860728 1140860736 1140860738 1140860750 1140860752 1140860758 1140860764 1140860776 1140860784 1140860790 1140860802 1140860806 1140860828 1140860830 1140860834 1140860836 1140860838 1140860840 1140860842 1140860846 1140860848 1140860862 1140860878 1140860882 1140860892 1140860904 1140860912 1140860918 1140860938 1140860942 1140860952 1140860954 1140860966 1140860972 1140860976 1140860982 1140860988 1140860994 1140861000 1140861002 1140861008 1140861010 1140861016 1140861022 1140861024 1140861034 1140861046 1140861068 1140861070 1140861088 1140861090 1140861106 1140861110 1140861114 1140861120 1140861128 1140861130 1140861136 1140861138 1140861166 1140861176 1140861190 1140861194 1140861202 1140861266 1140861268 1140861276 1140861282 1140861326 1140861384 1140864950 1140864952 1140866072 1140866074 1140866078 1140866084 1140866086 1140866090 1140866092 1140866094 1140866096 1140866102 1140866104 1140866108 1140866110 1140866116 1140866122 1140866128 1140866132 1140866136 1140866138 1140866140 1140866144 1140866146 1140866156 1140866158 1140866162 1140866164 1140866168 1140866182 1140866192 1140866194 1140866200 1140866202 1140866206 1140866210 1140866212 1140866220 1140866222 1140866226 1140866230 1140866232 1140866236 1140866244 1140866248 1140866262 1140866280 1140866282 1140866306 1140866308 1140866312 1140866318 1140866324 1140866328 1140866330 1140866332 1140866334 1140866340 1140866352 1140866354 1140866356 1140866360 1140866388 1140866390 1140866396 1140866400 1140866402 1140866404 1140866406 1140866408 1140866410 1140866412 1140866416 1140866418 1140866420 1140866422 1140866426 1140866438 1140866440 1140866442 1140866444 1140866446 1140866448 1140866450 1140866460 1140866466 1140866484 1140866506 1140866546 1140866554 1140866692 1140866704 1140866712 1140866724 1140866726 1140866738 1140866756 1140866758 1140866764 1140866766 1140866778 1140866782 1140866784 1140866798 1140866800 1140866802 1140866804 1140875808 1140879758 1140879760 1140879762 1140879778 1140879782 1140879786 1140879794 1140879798 1140879802 1140879806 1140879810 1140879818 1140879822 1140879824 1140879826 1140879830 1140879834 1140879842 1140879854 1140879866 1140888510 1140888512 1140888552 1140888556 1140888560 1140888578 1140888582 1140888586 1140888646 1140888686 1140888760 1140888762 1140909368 1140911698 1140916356 1140916362 1140917428 1140923572 1140923712 1140923718 1140926778 1140926780 1141145658 1141145660 1141145668 1141151016 1141151018 1141151382 1141152600 1141152998 1141153006 1141153026 1141153032 1141153328 1141156754 1141156808 1141156836 1141156846 1141157252 1141157254 1141164148 1141164154 1141164276 1141164280 1141165470 1141165476 1141166006 1141167822 1141167832 1141171152 1141171336 1141171344 1141172682 1141172686 1141172698 1141173888 1141180592 1141180598 1141187788 1141187790 1141190160 1141192064 1141193282 1141193346 1141194794 1141194800 1141194804 1141194808 1141194810 1141201038 1141201040

Corticosteroids	1140853854 1140854694 1140854700 1140854784 1140854788 1140854816 1140854834 1140854888 1140854916 1140854990 1140857672 1140857678 1140862572 1140868364 1140868370 1140873620 1140874790 1140874792 1140874794 1140874810 1140874814 1140874816 1140874822 1140874896 1140874930 1140874936 1140874940 1140874944 1140874950 1140874954 1140874956 1140874976 1140874978 1140875668 1140875684 1140876032 1140876036 1140876044 1140876046 1140876052 1140876058 1140876076 1140876104 1140876456 1140878562 1140879922 1140879934 1140881938 1140882152 1140882622 1140882624 1140882626 1140882630 1140882694 1140882708 1140882718 1140882722 1140882724 1140882728 1140882730 1140882732 1140882740 1140882742 1140882756 1140882758 1140882764 1140882766 1140882768 1140882774 1140882776 1140882778 1140882780 1140882782 1140882794 1140882800 1140882806 1140882808 1140882816 1140882818 1140882820 1140882822 1140882824 1140882826 1140882830 1140882832 1140882836 1140882840 1140882842 1140882844 1140882846 1140882848 1140882850 1140882852 1140882864 1140882888 1140882892 1140882894 1140882896 1140882898 1140882902 1140882904 1140882906 1140882908 1140882910 1140882914 1140882916 1140882918 1140882920 1140882926 1140882928 1140882932 1140882934 1140882938 1140883022 1140883026 1140883028 1140883030 1140883034 1140883038 1140883040 1140883044 1140883048 1140883052 1140883054 1140883056 1140883058 1140883060 1140883062 1140883064 1140884636 1140884640 1140884642 1140884646 1140884654 1140884660 1140884664 1140884672 1140884676 1140884696 1140884700 1140884704 1140884716 1140888074 1140888092 1140888098 1140888124 1140888130 1140888134 1140888142 1140888150 1140888166 1140888168 1140888172 1140888176 1140888178 1140888184 1140888194 1140909786 1140909894 1140910424 1140910634 1141151424 1141157294 1141157402 1141157418 1141162532 1141164086 1141167174 1141169844 1141173346 1141174512 1141174520 1141174548 1141174552 1141179072 1141179982 1141180342 1141181062 1141181554 1141181610 1141189464 1141191748 1141194840 1141195232 1141195280
Second generation atypical Psychotics	1140867420 1140867432 1140867444 1140927956 1140927970 1140928916 1141152848 1141152860 1141153490 1141167976 1141177762 1141195974 1141202024
Non-statin lipid-lowering therapies	1140865576 1140865576 1141157416 1140861924 1141157260 1140861926 1140861928 1140861936 1140861944 1140861922 1140861942 1140861946 1140861954 1140862026 1140862028 1141175908 1141168568 1141171548 1141201306 1140888590 1140861848 1140851880 1140851882 1140861856 1141157262 1140861858 1140926582 1140861866 1140861324 1140861868 1141188546 1140861876 1140861878 1140861884 1141181868 1140861892 1141162544 1141172214 1141182910 1140865752 1141157494 1141145830 1141192736 1141192740

Supplementary Table 3: ICD codes used to define incident and prevalent cases of cardiovascular disease

Cardiovascular event	ICD9	ICD10
Incident cardiovascular disease (all subtypes combined)	3900-4599	I* G45
Myocardial infarction	4100-4109, 4120-4129	I21, I22
Angina	4139	I20
Stroke	43- 4389	I6, G45
Transient ischaemic attack	4359	G45
Peripheral arterial disease	4439	I73.9
Type 1 diabetes	2500- 25011, 25013, 2504-25041, 25043, 2505-25051, 25053, 2506-25061, 25063, 2507-25071, 25073, 2509-25091, 25093	E10
Chronic kidney disease	5383, 5384, 5385	N183, N184, N185
Familial hypercholesterolaemia	2720	I78.0

Supplementary Table 4: International Standard for Classification of Education codes mapped to UK Biobank self-report highest qualification to estimate years of education

Qualification (As reported in UK Biobank)	ISCED	Years of education
**College or University degree**	5	20
**NVQ or HND or HNC or equivalent**	5	19
**Other prof. qual. e.g.: nursing, teaching**	4	15
**A levels/AS levels or equivalent**	3	13
**O levels/GCSEs or equivalent**	2	10
**CSEs or equivalent**	2	10
**None of the above**	1	7
**Prefer not to answer**	Excluded	

Supplementary Table 5: Proportion of missing data in QRISK3 variables

Variable	Female	Male
**% missing**		
QRISK	24%	22%
Age	0%	0%
BMI	0.5%	0.7%
Systolic blood pressure	9%	9%
Townsend deprivation index	0.1%	0.1%
Total cholesterol:HDL cholesterol	15%	13%
**% missing**		
Years of education	2%	2%
Ethnicity	0.5%	0.7%
Smoking	0%	0%
Family history of CVD	0%	0%
Statin (reported)	0%	0%
Statin type	0%	0%

Supplementary Table 6: Descriptive characteristics of UK Biobank participants in i) the full eligible (imputed) sample analysed ii) the eligible (imputed) sample who also have linked primary care data iii) participants with linked primary care data and a recorded QRISK score and iv) participants with complete data on QRISK3 variables

Variable		Imputed analysis sample (N = 472 097)		Primary care analysis sample (imputed) (N = 209 451)		Primary care analysis sample with recorded QRISK (N = 12 128)		Complete case analysis sample (N = 368 721)	
		Female (N = 261 147)	Males (N = 210 950)	Female (N = 117 038)	Males (N = 92 413)	Female (N = 7 338)	Male (N = 4 790)	Female (N = 201 532)	Male (N = 167 189)
**Continuous variables**		**Mean (SD)**							
QRISK*	QRISK3 (baseline)	6.87 (5.54)	12.98 (8.34)	6.94 (5.57)	13.11 (8.35)	6.21 (4.68)	11.44 (7.1)	6.84 (5.5)	12.97 (8.32)
	QRISK3 excluding 'non-validated' statin users	NA	NA	6.09 (4.98)	11.54 (7.82)	NA	NA	NA	NA
	Recoded value of QRISK in primary care	NA	NA	NA	NA	10.17 (6.94)	16.11 (9.2)	NA	NA
Age		56.23 (7.98)	56.44 (8.2)	56.26 (7.94)	56.5 (8.15)	56.28 (7.98)	56.45 (8.2)	56.28 (7.98)	56.45 (8.2)
BMI		27.02 (5.15)	27.75 (4.2)	27.14 (5.18)	27.86 (4.23)	26.96 (5.08)	27.74 (4.18)	26.96 (5.08)	27.74 (4.18)
Systolic blood pressure		135.14 (19.18)	140.94 (17.35)	135.46 (19.17)	141.31 (17.39)	135.15 (19.15)	141 (17.31)	135.15 (19.15)	141 (17.31)
Townsend deprivation index		-1.38 (3.2)	-1.31 (3.12)	-1.41 (2.95)	-1.36 (3.05)	-1.4 (2.99)	-1.34 (3.09)	-1.4 (2.99)	-1.34 (3.09)
Total cholesterol:HDL cholesterol		3.86 (1)	4.48 (1.15)	3.88 (1.01)	4.49 (1.15)	3.84 (1)	4.49 (1.15)	3.84 (1)	4.49 (1.15)
**Categorical variables**		**Percent of Sample (SE)**				**Frequency (%)**			
Years of education	≤7 years	14.21 (0.08)	13.83 (0.09)	15.29 (0.12)	14.67 (0.14)	1 034 (14)	601 (13)	32 785 (16)	26 874 (16)
	8-10 years	19.4 (0.09)	13.52 (0.09)	19.1 (0.13)	13.36 (0.13)	1 520 (21)	649 (14)	39 795 (20)	22 945 (14)
	11-13 years	6.06 (0.05)	5.27 (0.06)	5.81 (0.08)	5.05 (0.09)	436 (6)	285 (6)	11 729 (6)	8 449 (5)
	14-15 years	12.83 (0.07)	10.04 (0.08)	12.69 (0.11)	10.16 (0.12)	961 (13)	497 (10)	26 936 (13)	17 161 (10)
	16-19 years	12.88 (0.07)	19.67 (0.1)	13.13 (0.11)	20.17 (0.16)	911 (12)	944 (20)	25 653 (13)	32 940 (20)
	≥20 years	34.62 (0.11)	37.67 (0.12)	33.98 (0.16)	36.58 (0.19)	2 476 (34)	1 814 (38)	64 634 (32)	58 820 (35)
Ethnicity	White	94.96 (0.05)	94.7 (0.06)	95.75 (0.07)	95.33 (0.08)	7 026 (96)	4 600 (96)	190 903 (95)	158 386 (95)
	Indian	0.98 (0.02)	1.2 (0.03)	1.04 (0.03)	1.3 (0.04)	66 (1)	49 (1)	2 082 (1)	2 108 (1)
	Pakistani	0.23 (0.01)	0.42 (0.02)	26.52 (0.02)	0.46 (0.03)	21 (0)	11 (0)	462 (0)	717 (0)
	Other Asian	0.48 (0.02)	0.6 (0.02)	0.4 (0.02)	0.58 (0.03)	25 (0)	22 (0)	982 (0)	979 (1)
	Black Caribbean	10.73 (0.02)	0.81 (0.02)	0.77 (0.03)	0.64 (0.03)	55 (1)	18 (0)	2 464 (1)	1 408 (1)
	Black African	0.68 (0.02)	0.86 (0.02)	0.46 (0.02)	0.54 (0.03)	40 (1)	21 (0)	1 435 (1)	1 406 (1)
	Chinese	0.38 (0.01)	0.28 (0.01)	0.32 (0.02)	0.23 (0.02)	26 (0)	26 (0)	719 (0)	719 (0)

	Other	1.22 (0.02)	1.12 (0.03)	1.01 (0.03)	0.92 (0.04)	70 (1)	70 (1)	2 485 (1)	2 485 (1)
Smoking	Never	60.54 (0.11)	52.29 (0.13)	60.79 (0.16)	52.33 (0.19)	4 388 (60)	2 536 (53)	120 335 (60)	83 129 (50)
	Former	30.39 (0.1)	35.02 (0.12)	30.05 (0.15)	35.16 (0.19)	2 346 (32)	1 715 (36)	63 059 (31)	63 033 (38)
	Light (1-9/day)	1.66 (0.03)	1.29 (0.03)	1.59 (0.04)	1.24 (0.04)	128 (2)	57 (1)	3 287 (2)	2 056 (1)
	Moderate (10-19/day)	2.99 (0.04)	2.96 (0.04)	3.16 (0.06)	3.01 (0.07)	176 (2)	102 (2)	6 094 (3)	4 931 (3)
	Heavy (>20/day)	4.42 (4.42)	8.45 (0.07)	4.42 (0.07)	8.26 (0.11)	300 (4)	380 (8)	8 757 (4)	14 040 (8)
Type 2 diabetes	Control	99.07 (0.02)	98.31 (0.03)	99.09 (0.03)	98.30 (0.04)	7 329 (00)	4 780 (100)	199 700 (99)	164 395 (98)
	Case	0.93 (0.02)	1.69 (0.03)	0.91 (0.03)	1.70 (0.04)	9 (0)	10 (0)	1 832 (1)	2 794 (2)
Family history of CVD	Control	72.37 (0.1)	78.22 (0.11)	71.5 (0.15)	77.57 (0.16)	5 242 (71)	3 749 (78)	142 641 (71)	128 314 (77)
	Case	27.63 (0.1)	21.78 (0.11)	28.5 (0.15)	22.43 (0.16)	2 096 (29)	1 041 (22)	58 891 (29)	38 875 (23)
Cardiovascular risk (strata of QRISK score)	Low cardiovascular risk (<10%)	76.57 (0.09)	42.01 (0.01)	76.16 (0.13)	41.28 (0.17)	3 993 (54)	1 328 (28)	154 582 (77)	70 093 (42)
	Medium risk (≥10% - <20%)	20.70 (0.08)	39.59 (0.11)	21.08 (0.13)	39.98 (0.17)	2 685 (37)	1 964 (41)	41 579 (21)	66 488 (40)
	High risk (≥20%)	2.73 (0.03)	18.39 (0.09)	2.76 (0.05)	18.75 (0.13)	660 (9)	1 498 (31)	5 371 (3)	30 608 (18)
Statin (reported)	Control	90.27 (0.06)	82.99 (0.08)	90.14 (0.09)	82.39 (0.13)	NA	NA	181 903 (90)	138 619 (83)
	Case	9.73 (0.06)	17.01 (0.08)	9.86 (0.09)	17.61 (0.13)	NA	NA	19 629 (10)	28 570 (17)
Statin type	No statin	90.27 (0.06)	82.99 (0.08)	90.14 (0.09)	82.39 (0.13)	NA	NA	181 903 (90)	138 619 (83)
	Atorvastatin	1.64 (0.02)	2.87 (0.04)	1.68 (0.04)	2.9 (0.06)	NA	NA	19 629 (10)	28 570 (17)
	Fluvastatin	0.02 (0)	0.06 (0.01)	0.03 (0)	0.06 (0.01)	NA	NA	181 903 (90)	138 619 (83)
	Pravastatin	0.3 (0.01)	0.47 (0.01)	0.29 (0.02)	0.44 (0.02)	NA	NA	3 281 (2)	4 750 (3)
	Rosuvastatin	0.39 (0.01)	0.61 (0.02)	0.38 (0.02)	0.65 (0.03)	NA	NA	49 (0)	96 (0)
	Simvastatin	7.37 (0.05)	13.01 (0.07)	7.49 (0.08)	13.56 (0.11)	NA	NA	617 (0)	787 (0)
Statin (validated)	Control	NA	NA	97.62 (0.05)	95.40 (0.08)	6 345 (86)	3 878 (81)	NA	NA
	Case	NA	NA	2.38 (0.05)	4.60 (0.08)	993 (14)	912 (19)	NA	NA
Reported statin with no prescription*	Control	NA	NA	92.90 (0.08)	86.01 (0.13)	NA	NA	NA	NA
	Case	NA	NA	7.10 (0.08)	13.99 (0.13)	NA	NA	NA	NA
Non-statin lipid lowering therapy	Control (including statin users)	99.13 (0.02)	98.79 (0.02)	99.09 (0.03)	98.83 (0.04)	7 327 (100)	4 785 (100)	199 770 (99)	165 154 (99)
	Case	0.87 (0.02)	1.21 (0.02)	0.91 (0.03)	1.16 (0.04)	11 (0)	5 (0)	1762 (1)	2035 (1)
Incident CVD	Control	79.63 (0.08)	0.08 (73.66)	79.85 (0.13)	0.13 (73.57)	5 379 (82)	3 439 (80)	140 753 (79)	106 032 (74)
	Case	20.37 (0.08)	0.08 (26.34)	20.15 (0.13)	0.13 (26.43)	1 179 (18)	885 (20)	36 401 (21)	38 171 (26)

Derived QRISK3 variable from baseline measured in UK Biobank for the full analysis sample and primary care analysis sample, recorded QRISK or QRISK2 scores in primary care data for the primary care analysis sample with recorded QRISK.
*Proportion of individuals excluding individuals with validated prescriptions

Supplementary Table 7: Odd ratio for i) statin use and ii) incident cardiovascular disease per unit increase in QRISK3 score and unit increase in years of education, adjusted for date of baseline assessment

Exposure	Outcome	Females		Males	
		Complete Case Odds ratio (95% CI) (N = 201 532)	Imputed sample Odds ratio (95% CI) (N = 261 147)	Complete Case Odds ratio (95% CI) (N = 167 189)	Imputed sample Odds ratio (95% CI) (N = 210 950)
**QRISK3**	Statins (any)	1.123 (1.120, 1.125)	1.124 (1.122 1.126)	1.070 (1.069, 1.072)	1.072 (1.070, 1.073)
	Incident cardiovascular event	1.143 (1.140, 1.146)	1.119 (1.116, 1.122)	1.088 (1.086, 1.090)	1.082 (1.080, 1.084)
**Education**	Statins (any)	0.929 (0.927, 0.932)	0.929 (0.926, 0.931	0.958 (0.955, 0.960	0.958 (0.956, 0.960)
	Incident cardiovascular event	0.949 (0.946, 0.951)	0.949 (0.946, 0.951)	0.956 (0.954, 0.958)	0.948 (0.945, 0.951)

Supplementary Table 8: Mean difference in QRISK3 score per unit increase in educational attainment

Outcome	Females		Males	
	Complete Case Mean difference (95% CI) (N = 201 532)	Imputed Sample Mean difference (95% CI) (N = 261 147)	Complete Case Mean difference (95% CI) (N = 167 189)	Imputed Sample Mean difference (95% CI) (N = 210 950)
**QRISK3**	-0.292 (-0.297, -0.288)	-0.296 (-0.300, -0.292)	-0.341 (-0.349, -0.333)	-0.346 (-0.354, -0.340)

Supplementary Table 9: Percent of participants reporting statin use in low, medium and high cardiovascular risk groups, stratified by years of education and the association between education and statin use stratified by cardiovascular risk, adjusted by date of baseline assessment centre

10-year cardiovascular risk	Years of Education	Females (N = 261 147)			Males (N = 210 950)		
		Percent of participants within strata of cardiovascular risk per years of education (SE)	Percent of self-reported statin users	Odds ratio for statin use (95% CI)	Percent of participants within strata of cardiovascular risk per years of education (SE)	Percent of self-reported statin users	Odds ratio for statin use (95% CI)
Low risk <10	All years		6.39 (0.06)			6.81 (0.09)	
	≤7 years	11.46 (0.07)	12.24 (0.22)	Reference	7.43 (0.09)	9.88 (0.04)	Reference
	8-10 years	19.64 (0.09)	6.63 (0.13)	0.51 (0.48, 0.54)	14.88 (0.12)	6.22 (0.22)	0.60 (0.54, 0.68)
	11-13 years	6.21 (0.05)	5.52 (0.21)	0.42 (0.38, 0.46)	5.66 (0.08)	7.03 (0.37)	0.69 (0.60, 0.79)
	14-15 years	12.79 (0.08)	7.26 (0.16)	0.56 (0.53, 0.60)	8.76 (0.10)	8.05 (0.31)	0.80 (0.71, 0.90)
	16-19 years	13.91 (0.08)	5.62 (0.14)	0.43 (0.40, 0.46)	21.32 (0.14)	6.67 (0.19)	0.65 (0.59, 0.72)
	≥20 years	36.00 (0.10)	4.54 (0.01)	0.34 (0.32, 0.36)	41.95 (0.17)	6.26 (0.13)	0.61 (0.55, 0.67)
	All years		19.60 (0.18)			22.40 (0.15)	
Medium risk (≥10 & <20)	≤7 years	31.72 (0.21)	23.26 (0.33)	Reference	19.18 (0.14)	26.58 (0.36)	Reference
	8-10 years	19.03 (0.17)	19.26 (0.41)	0.79 (0.74, 0.84)	12.67 (0.12)	22.94 (0.42)	0.82 (0.78, 0.87)
	11-13 years	4.67 (0.09)	16.80 (0.77)	0.67 (0.60, 0.75)	4.84 (0.08)	21.25 (0.66)	0.75 (0.69, 0.81)
	14-15 years	14.46 (0.09)	18.69 (0.45)	0.76 (0.71, 0.81)	11.44 (0.11)	22.78 (0.43)	0.82 (0.77, 0.87)
	16-19 years	9.09 (0.13)	18.58 (0.56)	0.75 (0.69, 0.82)	18.59 (0.14)	21.69 (0.34)	0.76 (0.73, 0.81)
	≥20 years	21.02 (0.18)	16.08 (0.35)	0.63 (0.59, 0.67)	33.27 (0.17)	20.23 (0.25)	0.70 (0.67, 0.73)
	All years		28.42 (0.56)			28.72 (0.24)	
High risk (≥20%)	≤7 years	43.65 (0.61)	30.26 (0.86)	Reference	30.78 (0.24)	31.35 (0.43)	Reference
	8-10 years	16.21 (0.45)	28.61 (0.01)	0.92 (0.78, 1.07)	12.37 (0.17)	29.27 (0.67)	0.91 (0.84, 0.98)
	11-13 years	3.80 (0.23)	27.61 (2.78)	0.88 (0.66, 1.17)	4.20 (0.11)	25.64 (1.11)	0.76 (0.67, 0.85)
	14-15 years	12.69 (0.41)	28.61 (1.56)	0.92 (0.78, 1.09)	10.97 (0.16)	29.39 (0.71)	0.91 (0.84, 0.99)
	16-19 years	8.24 (0.34)	28.51 (1.96)	0.91 (0.74, 1.12)	17.22 (0.20)	28.17 (0.56)	0.91 (0.80, 0.92)
	≥20 years	15.42 (0.44)	23.03 (1.29)	0.69 (0.58, 0.81)	24.47 (0.22)	25.76 (0.47)	0.76 (0.71, 0.81)

SE = standard error; CI = confidence interval

Supplementary Table 10: Odds ratio for Atorvastatin (case) use compared with Simvastatin (control) use per unit increase in QRISK3 score and by strata of educational attainment (not accounting for interactions), adjusted for date of baseline assessment

Exposure		Females		Males	
		**Complete Case Odds ratio (95% CI)** (N = 18 180)	**Imputed sample Odds ratio (95% CI)** (N = 23 538)	**Complete Case Odds ratio (95% CI)** (N = 26 633)	**Imputed sample Odds ratio (95% CI)** (N = 33 499)
**QRISK3**		1.023 (1.017, 1.029)	1.0249 (1.020, 1.030)	1.017 (1.013, 1.021)	1.016 (1.013, 1.020)
**Education**	All years	1.001 (0.997, 1.006)	0.994 (0.988, 1.001)	1.004 (0.998, 1.010)	1.001 (0.996, 1.006)
	≤7 years	Baseline		Baseline	
	8-10 years	1.033 (0.93, 1.15)	0.992 (0.901, 1.091)	1.033 (0.926, 1.153)	0.990 (0.899, 1.090)
	11-13 years	1.16 (0.926, 1.394)	1.079 (0.919, 1.267)	0.992 (0.843, 1.167)	1.001 (0.868, 1.153)
	14-15 years	1.139 (1.011, 1.284)	1.071 (0.965 1.190)	1.003 (0.895, 1.124)	0.980 (0.886, 1.084)
	16-19 years	0.989 (0.863, 1.133)	0.930 (0.825, 1.048)	1.026 (0.930, 1.132)	0.990 (0.907, 1.079)
	≥20 years	0.940 (0.842, 1.049)	0.911 (0.829, 1.002)	1.070 (0.981, 1.167)	1.018 (0.943, 1.099)

Note: Atorvastatin is generally regarded as more efficacious than Simvastatin. Simvastatin is available to purchase over the counter

Supplementary Table 11: Odd ratio for i) statin use and ii) Atorvastatin use (case) compared with Simvastatin (control) use per unit increase in QRISK3 score stratified by educational attainment in the complete case sample to test for evidence of a multiplicative interaction

Outcome	Years of education	Females		Males	
		Complete Case Odds ratio (95% CI) (N = 261 147)	P Value for interaction	Complete Case Odds ratio (95% CI) (N = 210 950)	P Value for interaction
Statins (self-report)	≤7 years	1.068 (1.064, 1.073)	7.83×10^{-105}	1.042 (1.039, 1.045)	7.40×10^{-66}
	8-10 years	1.123 (1.117, 1.129)		1.078 (1.073, 1.082)	
	11-13 years	1.131 (1.119, 1.144)		1.064 (1.057, 1.072)	
	14-15 years	1.119 (1.112, 1.127)		1.061 (1.056, 1.066)	
	16-19 years	1.140 (1.132, 1.149)		1.075 (1.071, 1.079)	
	≥20 years	1.141 (1.135, 1.147)		1.079 (1.076, 1.082)	
		Complete Case Odds ratio (95% CI) (N = 18 180)		Complete Case Odds ratio (95% CI) (N = 26 633)	
Statin type (atorvastatin vs simvastatin)	≤7 years	1.021 (1.011, 1.031)	0.733	1.019 (1.012, 1.027)	0.061
	8-10 years	1.029 (1.015, 1.042)		1.012 (1.002, 1.023)	
	11-13 years	1.039 (1.014, 1.065)		1.015 (0.997, 1.033)	
	14-15 years	1.023 (1.008, 1.039)		1.031 (1.019, 1.043)	
	16-19 years	1.017 (0.988, 1.035)		1.023 (1.014, 1.032)	
	≥20 years	1.024 (1.010, 1.038)		1.012 (1.005, 1.019)	

Analyses adjusted for date of baseline assessment centre

Supplementary Table 12: Odds ratio for statin use per unit increase in QRISK3 score stratified by educational attainment in the complete case sample to test for evidence of an interaction, excluding participants on non-statin lipid-lowering therapies

		Females		Males	
**Outcome**	**Years of education**	**Odds ratio using imputed data (95% CI)** (N = 258 863)	**P Value for interaction**	**Odds ratio using imputed data (95% CI)** (N = 208 400)	**P Value for interaction**
Statins (self-report)	≤7 years	1.071 (1.067, 1.077)	$117x10^{-82}$	1.044 (1.041, 1.047)	$5.15x10^{-46}$
	8-10 years	1.126 (1.121, 1.132)		1.078 (1.074, 1.082)	
	11-13 years	1.134 (1.123, 1.145)		1.068 (1.062, 1.075)	
	14-15 years	1.120 (1.114, 1.127)		1.065 (1.061, 1.070)	
	16-19 years	1.143 (1.135, 1.151)		1.077 (1.073, 1.080)	
	≥20 years	1.145 (1.139, 1.150)		1.081 (1.078, 1.084)	
		**Odds ratio using complete case data (95% CI)** (N = 199 770)	**P Value for interaction**	**Odds ratio using complete case data (95% CI)** (N = 165 154)	**P Value for interaction**
Statins (self-report)	≤7 years	1.070 (1.065, 1.075)	$5.17x10^{-26}$	1.043 (1.040, 1.046)	$1.04x10^{-16}$
	8-10 years	1.125 (1.119, 1.132)		1.079 (1.073, 1.082)	
	11-13 years	1.131 (1.118, 1.143)		1.065 (1.057, 1.072)	
	14-15 years	1.121 (1.113, 1.128)		1.063 (1.058, 1.068)	
	16-19 years	1.144 (1.136, 1.153)		1.076 (1.072, 1.080)	
	≥20 years	1.143 (1.137, 1.149)		1.080 (1.076, 1.083)	

Analyses adjusted for date of baseline assessment centre
In the imputation sample 2 284 females and 2 550 males were excluded for use of non-statin lipid lowering therapies.
In the complete-case sample, 1 726 females and 2035 males were excluded for use of non-statin lipid lowering therapies.

Supplementary Table 13: Pairwise correlation for QRISK3 scores derived from baseline measures in UK Biobank including all variables and excluding i) family history of CVD and iii) systolic blood pressure variability

QRISK3 score	Pairwise correlation with complete score
**Female**	
Excluding reported family history of any cardiovascular disease at any age	0.9799
Excluding systolic blood pressure from two baseline measures of systolic blood pressure	0.9991
**Male**	
Excluding reported family history of any cardiovascular disease at any age	0.9736
Excluding systolic blood pressure from two baseline measures of systolic blood pressure	0.9984

Additional Figures

Supplementary Figure 1: Schematic of primary and secondary analyses carried out

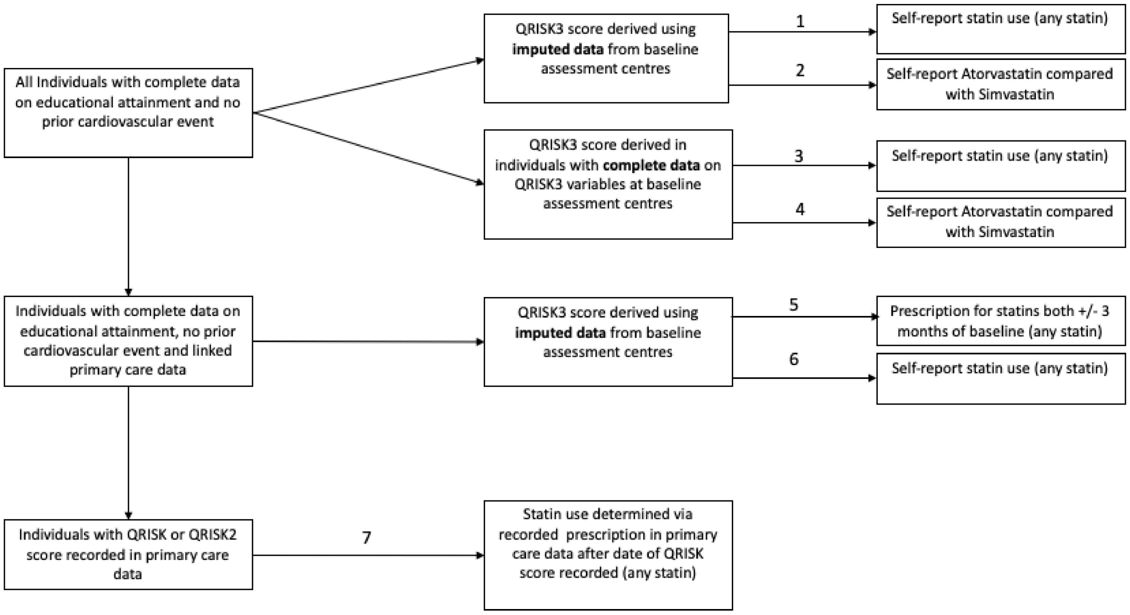

Supplementary Figure 2: Prevalence of statin use by decile of QRISK3 score in females and males with complete data

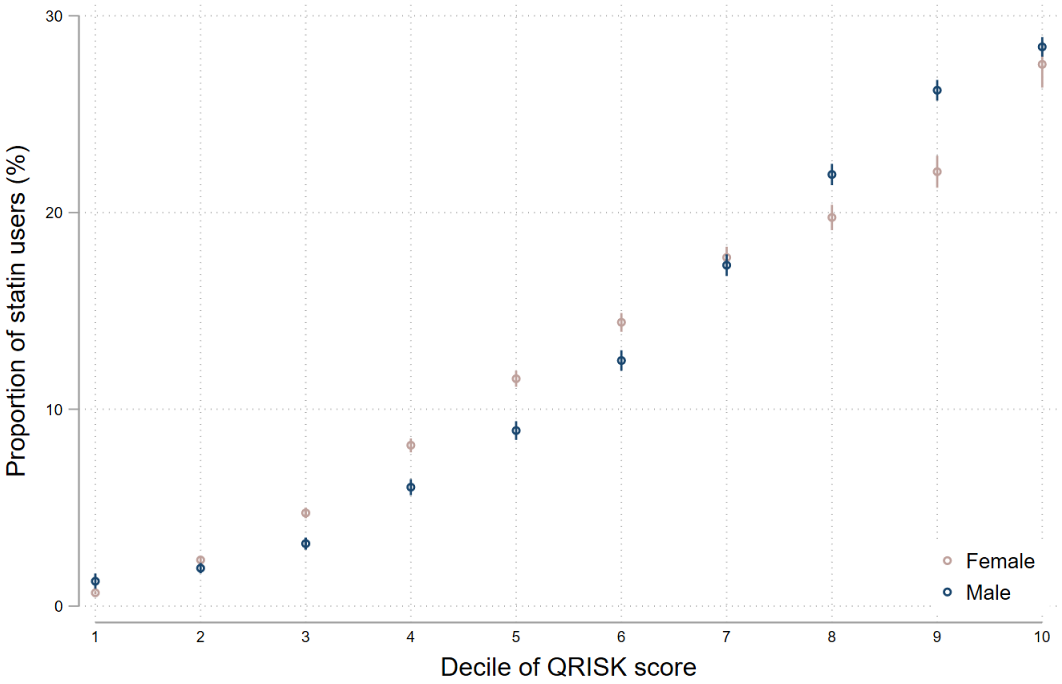

Supplementary Figure 3: Mean concentration of low-density lipoprotein cholesterol by years of education in females and males stratified by self-report statin use

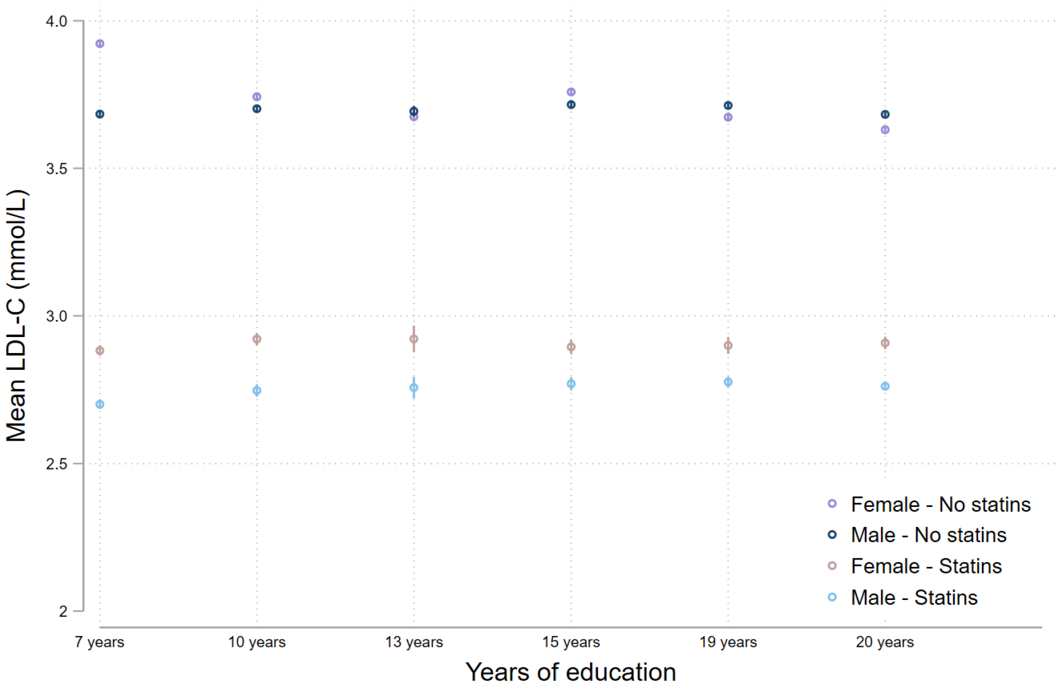

Supplementary Figure 4: Mean and median values of QRISK3 score on those with complete data, by years of education for females and males

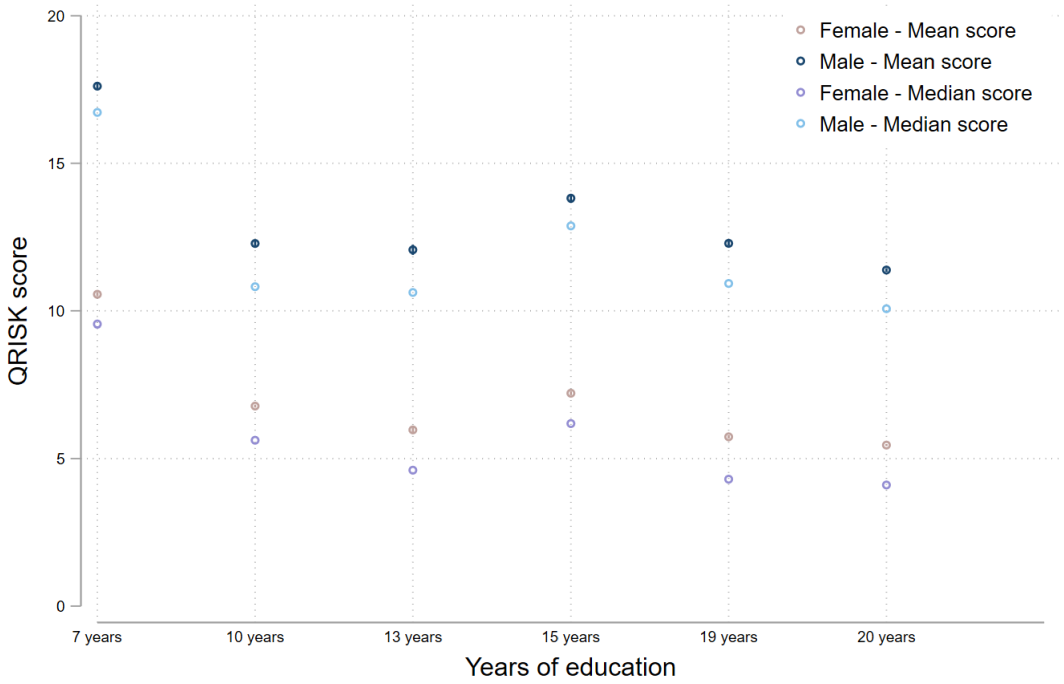

Supplementary Figure 5: Prevalence of statin prescribing by years of education in females and males with complete data

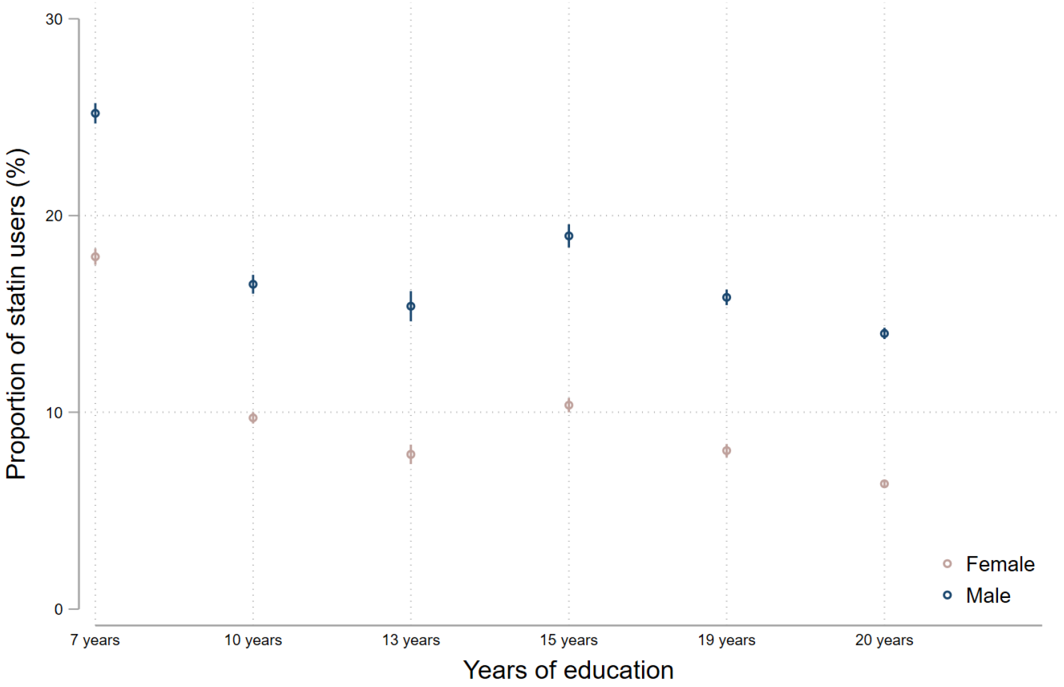

Supplementary Figure 6: Odds ratio for statin use per year unit increase in educational attainment (all years) and per strata of educational attainment

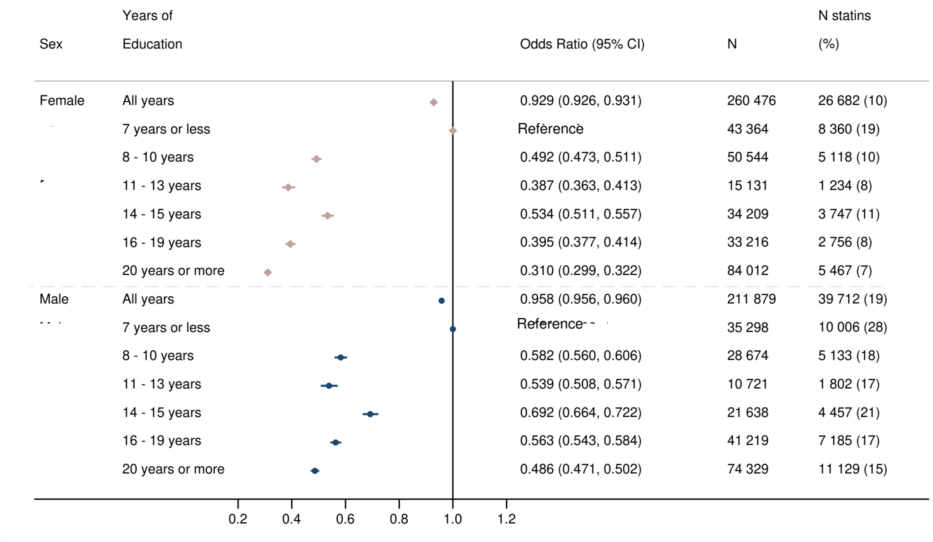

Analyses adjusted for date of baseline assessment centre

Supplementary Figure 7: Odds ratio for Atorvastatin prescribing (case) compared to Simvastatin (control), per unit increase in QRISK3 score with no education interaction and stratified by years of education in females and males to test for evidence of an interaction

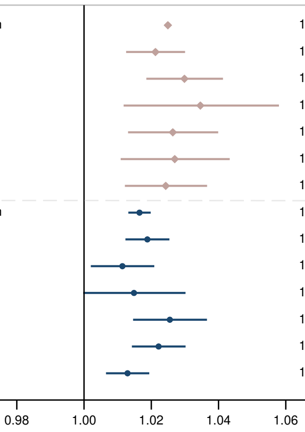

Sex	Years of Education	Odds Ratio (95% CI)	N	[illegible] (%)
Female	No education interaction	1.025 (1.025, 1.025)	24 652	26 682 (108)
	7 years or less	1.021 (1.013, 1.030)	43 364	8 360 (19)
	8 - 10 years	1.030 (1.019, 1.041)	50 544	5 118 (10)
	11 - 13 years	1.035 (1.012, 1.058)	15 131	1 234 (8)
	14 - 15 years	1.026 (1.013, 1.040)	34 209	3 747 (11)
	16 - 19 years	1.027 (1.011, 1.043)	33 216	2 756 (8)
	20 years or more	1.024 (1.012, 1.037)	84 012	5 467 (7)
Male	No education interaction	1.016 (1.013, 1.020)	211 879	39 712 (19)
	7 years or less	1.019 (1.012, 1.025)	35 298	10 006 (28)
	8 - 10 years	1.011 (1.002, 1.021)	28 674	5 133 (18)
	11 - 13 years	1.015 (1.000, 1.030)	10 721	1 802 (17)
	14 - 15 years	1.026 (1.015, 1.037)	21 638	4 457 (21)
	16 - 19 years	1.022 (1.014, 1.030)	41 219	7 185 (17)
	20 years or more	1.013 (1.007, 1.019)	74 329	11 129 (15)

Analyses adjusted for date of baseline assessment centre
P value for interaction in females = 0.418 and males = 0.894

Supplementary Figure 8: Odds ratio for self-report statin use per unit increase in baseline QRISK3 score with no education interaction and stratified by years of education to test for evidence of an interaction in the subsample of females and males with linked primary care data

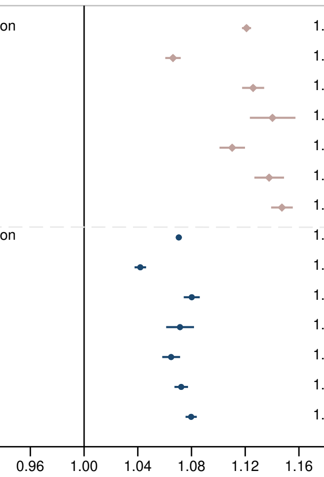

Sex	Years of Education	Odds Ratio (95% CI)	N	N statins (%)
Female	No education interaction	1.121 (1.118, 1.124)	117 038	11 538 (10)
	7 years or less	1.066 (1.060, 1.072)	20 552	3 621 (18)
	8 - 10 years	1.126 (1.118, 1.134)	22 412	2 217 (10)
	11 - 13 years	1.140 (1.123, 1.157)	6 545	506 (8)
	14 - 15 years	1.110 (1.101, 1.120)	15 226	1 583 (10)
	16 - 19 years	1.138 (1.127, 1.149)	15 183	1 218 (8)
	20 years or more	1.147 (1.139, 1.155)	37 120	2 393 (6)
Male	No education interaction	1.071 (1.068, 1.073)	92 413	16 273 (18)
	7 years or less	1.042 (1.038, 1.046)	15 828	3 994 (25)
	8 - 10 years	1.080 (1.074, 1.086)	12 298	2 073 (17)
	11 - 13 years	1.071 (1.061, 1.082)	4 485	702 (16)
	14 - 15 years	1.065 (1.058, 1.071)	9 565	1 878 (20)
	16 - 19 years	1.072 (1.067, 1.077)	18 395	2 964 (16)
	20 years or more	1.080 (1.076, 1.084)	31 842	4 662 (15)

Analyses adjusted for date of baseline assessment centre
P value for interaction in females = 4.727×10^{-48} and males = 1.026×10^{-20}

Supplementary Figure 9 : Risk difference for self-report statin use per unit increase in baseline QRISK3 score with no education interaction and stratified by years of education in females and males to test for an interaction on the additive scale

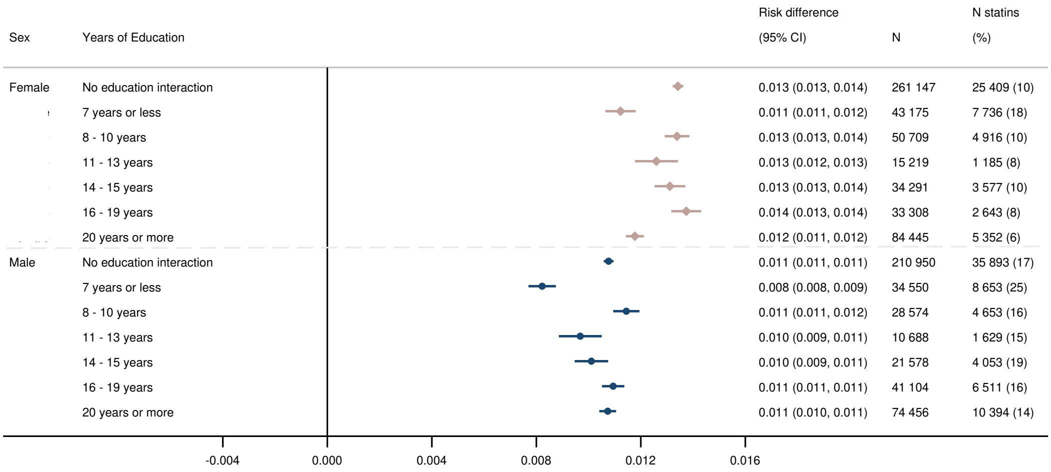

Analyses adjusted for date of baseline assessment centre

P value for interaction in females = 0.062 and males = 1.017×10^{-6}
